# Non-Destructive Evaluation of the Cutting Surface of Hardwood Finger Joints

**DOI:** 10.3390/s22103855

**Published:** 2022-05-19

**Authors:** Hannes Stolze, Michael Gurnik, Tim Koddenberg, Jonas Kröger, Robert Köhler, Wolfgang Viöl, Holger Militz

**Affiliations:** 1 Wood Biology and Wood Products, Faculty of Forest Sciences and Forest Ecology, University of Goettingen, Buesgenweg 4, 37077 Goettingen, Germany; michael.gurnik@uni-goettingen.de (M.G.); tim.koddenberg@uni-goettingen.de (T.K.); holger.militz@uni-goettingen.de (H.M.); 2 Department for Cutting and Joining Manufacturing Processes, Institute of Production Technology and Logistics, University of Kassel, Kurt-Wolters-Straße 3, 34125 Kassel, Germany; jonas.kroeger@unity-mail.de; 3 Laboratory of Laser and Plasma Technologies, Faculty of Engineering and Health, University of Applied Sciences and Arts, Von-Ossietzky-Straße 99, 37085 Goettingen, Germany; robert.koehler@hawk.de (R.K.); wolfgang.vioel@hawk.de (W.V.)

**Keywords:** finger-jointing, hardwood, wood characterization, non-destructive evaluation, cutting surface, wettability, roughness, adhesive penetration

## Abstract

In this study, the surface parameters wettability, roughness, and adhesive penetration, which are important for wood bonding, were investigated and evaluated utilizing non-destructive methods after different mechanical processing. For this purpose, beech and birch finger joints were prepared with different cutting combinations (three cutters with different sharpness levels and two feed rates) in an industrial process. Effects and interactions on the surface parameters resulting from the different cutting combinations were evaluated using three Full Factorial Designs. The various cutting parameters had a predominantly significant influence on the surface parameters. The effects and identified interactions highlight the complexity of the cutting surface and the importance of wood bonding. In this respect, a new finding is that with sharper cutters, higher contact angles of the adhesives occur. The methods (contact angle measurement, laser scanning microscopy, and brightfield microscopy) used were well suited to make effects visible and quantifiable, which can be of interest for the quality control of the wood processing industry. The results can help to better understand and evaluate the design of wood surfaces via machining and the bonding of hardwoods. Possibly the results can contribute to further standardizing the production of load-bearing hardwood finger joints and making them more efficient.

## 1. Introduction

There are high and increasing hardwood stocks in the German forest [1], and so far, a high proportion of these have been used for low-value purposes. In Germany, higher-value wood use is almost exclusively dominated by softwood, which is declining in volume [2,3]. Recently, new opportunities have emerged to use hardwoods such as beech and birch in high-grade engineered wood products (EWPs) in the building sector. Intensive research has resulted in national and European building authority approvals for various structural hardwood products [4,5,6,7,8]. In the timber industry, there is much less experience with EWPs based on hardwood compared to softwood. This also affects the finger-jointing, which is a mechanically efficient and longitudinal joint necessary to produce glued EWPs such as glued laminated timber [9,10]. Finger-jointing requires precise knowledge of the wood species-specific properties and adjustments in production technology, especially with anatomically complex hardwoods. The density-related high strength of hardwoods makes efficient finger-jointing necessary, and, in addition, the demands on machines and tools increase when processing hardwoods [11,12,13]. For high-quality bonding, the wood surface plays a crucial role [14]. In the finger-jointing process, it can be designed during cutting. The cutter sharpness, cutter feed rate, and rotational speed are significant influencing factors [15]. In the best case, non-destructive methods for the characterization of the cutting surface are available, which allow an evaluation of the cutting quality and adjustments in the production process. The objective of this study was to identify suitable methods to show the effects of the finger joint cutting on the quality of the wood surface and to provide surface analysis options for the quality control of the production process. The identified methods are based on previous investigations on wood wetting, wood roughness, and adhesive penetration into the porous wood structure and cell wall. Wetting analyses have been established as an important method for investigating wood bonding and adhesives. The porous anatomical structure and different surface chemistry of wood species make wood wetting analysis more challenging than more homogeneous materials such as plastics [16,17,18,19,20]. Measurements of wood roughness have proven to be particularly suitable for evaluating machined wood surfaces following production processes, such as bonding. In this respect, the wood anatomical structure, depending on the wood section and measurement direction, has a significant influence in addition to the machining process [21,22,23,24,25,26]. Various methods have been developed, and technologies from other research disciplines have been tested to describe the penetration of adhesives or other liquids into the wood structure. Especially the penetration into hardwoods is complex. Knowledge about the formation of adhesive joints is important for assessing the bonding quality and performance of bonded wood products. The influence of the wood surface on adhesive penetration has received little attention so far [27,28,29,30,31,32]. The identified methods of this study were used to characterize the cutting surface of beech and birch finger joints regarding subsequent further processing. Based on the empirical data of this study, comparisons of the cutting parameters used were made.

## 2. Materials and Methods

**Figure 1:**
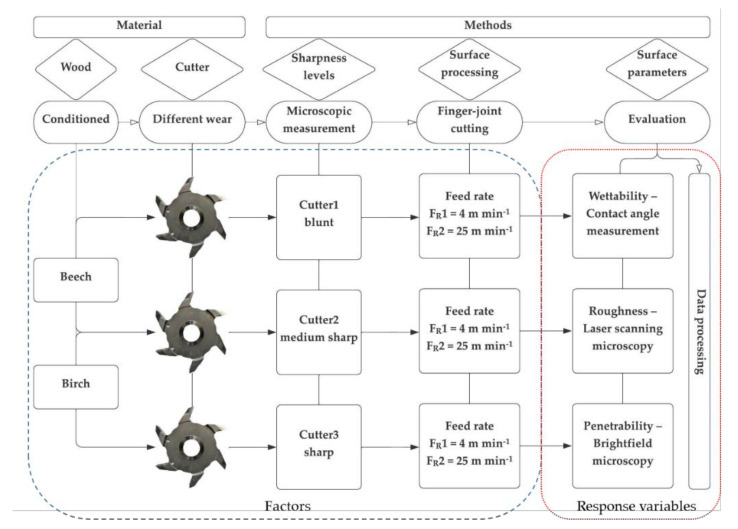
Overview chart of the material and methods in chronological order, the factors and the response variables of this study are highlighted.

The workflow of this study, considering the examined materials, used methods, and evaluated parameters are illustrated in Figure 1.

### 2.1. Wood and Finger Joint Cutter

In this study, the diffuse-porous wood species beech (*Fagus sylvatica*, L.; origin Germany) and birch (*Betula pendula*, L.; origin Latvia) were investigated. Two lamellas (side boards, flatsawn grain) with the dimensions 800 × 120 × 30 mm^3^ (long. × tang. × rad.) were cut from each wood species and conditioned in a climate at 20 °C and 65% relative humidity until the equilibrium moisture content of the wood was reached. The resulting average wood-moisture content of the beech and birch wood was 11.8% and 11.4%. The average density of the beech was 0.73 g cm^−3^ and that of the birch 0.58 g cm^−3^. The lamellae were as free as possible from wood characteristics such as knots, cracks, fiber deviations, or wood staining. The predominantly lying annual rings were aligned parallel in relation to the broad side of the lamella.

Three finger joint cutters recommended for the production of glued laminated timber and laminated beams according to EN 14080:2013 [33] were used. The cutters had different levels of wear. Cutter 1 (Cu1, blunt) had a long operating time, Cutter 2 (Cu2, medium sharp) had a medium operating time, and Cutter 3 (Cu3, sharp) was new.

### 2.2. Cutter Sharpness Levels and Surface Processing

**Figure 2:**
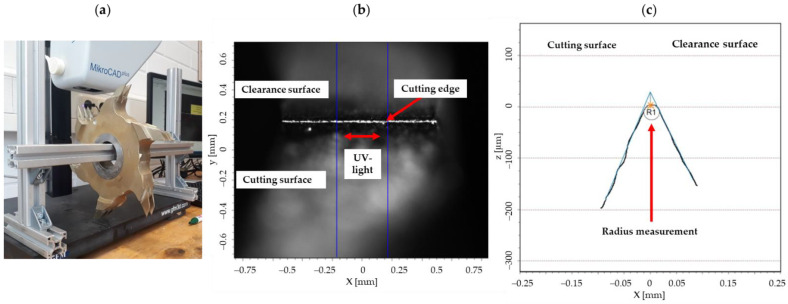
Microscopic measurement of the cutting edges to determine the degree of sharpness; (**a**) shows one of the examined cutter segments, (**b**) illustrates the components of the cutting edge and the measuring range, (**c**) shows the software-based determination of the cutting-edge radius as an indicator for the cutter wear.

The finger joints were cut using an Ultra TT finger-jointing line (Weinig Grecon GmbH & Co. KG, Alfeld, Germany). The line was equipped with three different sharp cutters; Cu1 (blunt), Cu2 (medium sharp), and Cu3 (sharp), one after the other. Before the cutting process was started, as a key indicator for the wear of cutters, the cutting-edge radius of seven cutting edges per cutter was measured microscopically. A Sub-Micron MikroCAD plus (GFMesstechnik GmbH, Teltow, Germany) structured UV-light 3D scanner was used for this measurement (Figure 2). The cutting-edge radius was measured with 100 light strips in each case, of which the 20 largest measured deviations were eliminated by the software.

**Figure 3:**
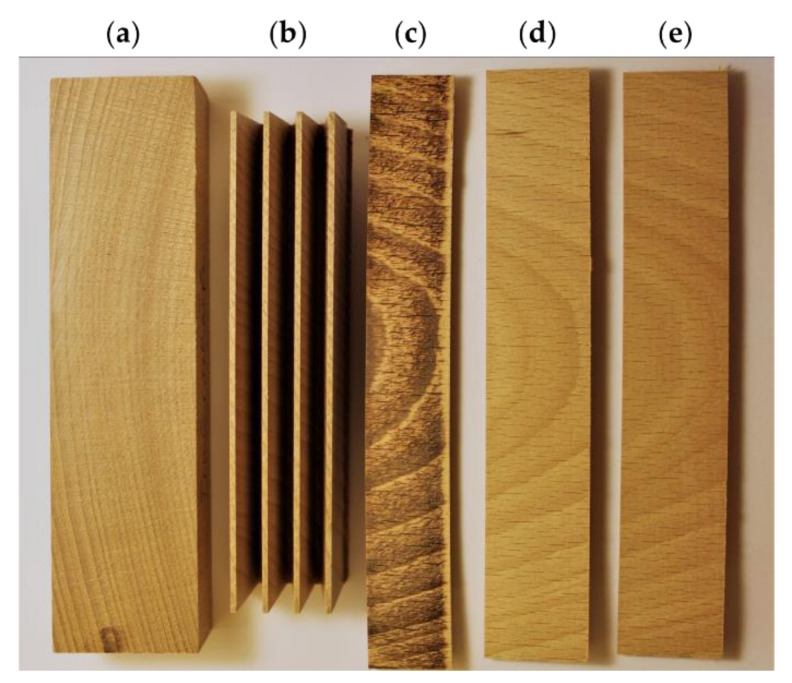
Surface processing of beech finger joints: (**a**) cross-section lamella; (**b**) horizontally cut finger joints; exemplary finger joints cut with (**c**) Cu1 (blunt) and F_R_1 (4 m min^−1^), (**d**) Cu2 (medium sharp) and F_R_1 (4 m min^−1^), (**e**) Cu3 (sharp) and F_R_1 (4 m min^−1^) for further surface evaluation.

The lamellae were cut at two different feed rates. A slower feed rate of 4 m min^−1^ (F_R_1) and a faster feed rate of 25 m min^−1^ (F_R_2) were used. The cutting was done in a horizontal direction and thus parallelled the alignment of the annual rings. For each wood species, six different combinations of surface processing resulting from the three cutters (blunt, medium sharp, and sharp) and two feeds rates (4 m min^−1^ and 25 m min^−1^) were examined. After each cutting process, the finger joints were separated from the lamella (shown exemplarily for several combinations of beech in Figure 3) and further investigated.

### 2.3. Surface Parameters and Data Processing

#### 2.3.1. Evaluation of Wettability

Using the method of contact angle measurement, the wettability of the wood surfaces resulting from the six different combinations of surface processing was evaluated for both wood species using the DSA 100 E (Krüss GmbH, Hamburg, Germany). Three test liquids and two drop generation methods were used (Table 1).

**Figure 4:**
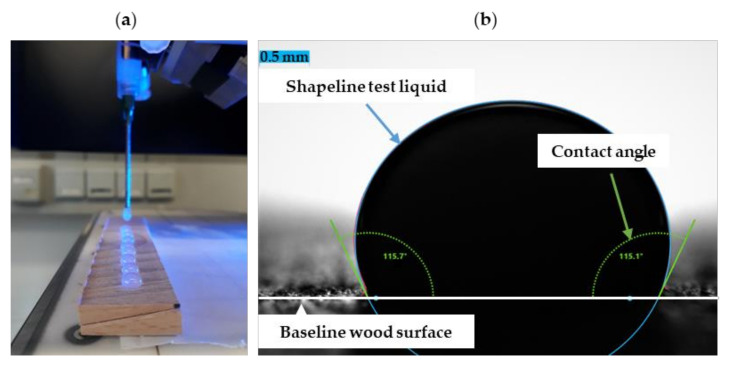
Application of adhesive on a freshly processed wood surface in the tangential direction (**a**) and contact angle measurement by the sessile-drop method, (**b**) shows the contour of an adhesive droplet after application to the wood surface and the calculated contact angle using the ADVANCE robust fitting algorithm, according to Krüss GmbH software-based range of the contact angle is 0 to 180° and resolution is 0.01°.

The test liquids were applied drop-shaped to the freshly cut, tangential surfaces of wood, ideally, in equal parts to the early- and latewood to minimize the effects of structural and chemical variations of the wood samples and measured in a tangential direction. For each combination, at least 12 measurements were carried out on dosed, sessile drops using drop contour analysis (Figure 4). The surfaces were leveled without inclination. The contact angles were measured using the static sessile drop method (without external interference during the measurement), keeping the time influence as small as possible through predefined measuring times. The water drop contours were measured 1 s after application because of rapid penetration of the low viscous water into end-grain wood. The higher viscous adhesive resin drops were measured 5 s after application and droplet stabilization as was carried out in other studies [34,35,36]. The contact angle results from the angle between the determined drop shape function (Fitmethod Ellipse Tang.-1) and the wood surface. The projection of the wood surface in the drop image is called the baseline. The calculation of the contact angle to the right and left of the drops and the mean was done automatically by the software ADVANCE (Krüss GmbH, Hamburg, Germany).

#### 2.3.2. Evaluation of Roughness

Surface morphology characterization was carried out on the tangentially oriented surfaces (16 to 21 × 120 mm^2^, long. × tang.) of the cut beech and birch finger joints and measured in a tangential direction. Measurements were performed with a confocal 3D laser scanning microscope (LSM, microscope: VK-X110, control unit: VK-X100; Keyence Corporation, Osaka, Japan) equipped with a red laser (658 nm). Initially, different magnifications and measuring ranges were tested. For the serial measurements, a 10× magnification measuring quality “superfine” (2043 × 1536 px) and a resulting resolution of 0.434 µm were used. A field of view of 1350 × 1012 µm^2^ was scanned for each image. Technically, the vertical linear scale of the LSM was limited to 0.005 µm. For each cutting combination, two areas were scanned across the annual rings along the tangential surface (12 × 1 and 29 × 1 images), and the image data were automatically merged to form an overall image. The topographic data were analyzed using the software VK-H1XAD, module ISO 25178 (Keyence Corporation, Osaka, Japan), following normative specifications (EN ISO 25178-1:2016; [37]). For each cutting combination, 24 rectangular cross-sections (1000 × 1250 µm^2^, long. × tang.) were defined, if possible, separately in the early- and latewood sections. An automated, linear inclination correction of the measuring range was carried out as an F-operation. Long-wavelength components were removed from the SF surface by applying a double Gaussian filter (EN ISO 16610-1:2015; [38]). The surface-related roughness parameter arithmetic mean height Sa (µm) was calculated software-based to quantify the surface properties. It quantifies, for each point of the surface, the difference in height to the arithmetic mean height of the total surface.

#### 2.3.3. Evaluation of Penetrability

**Figure 5:**
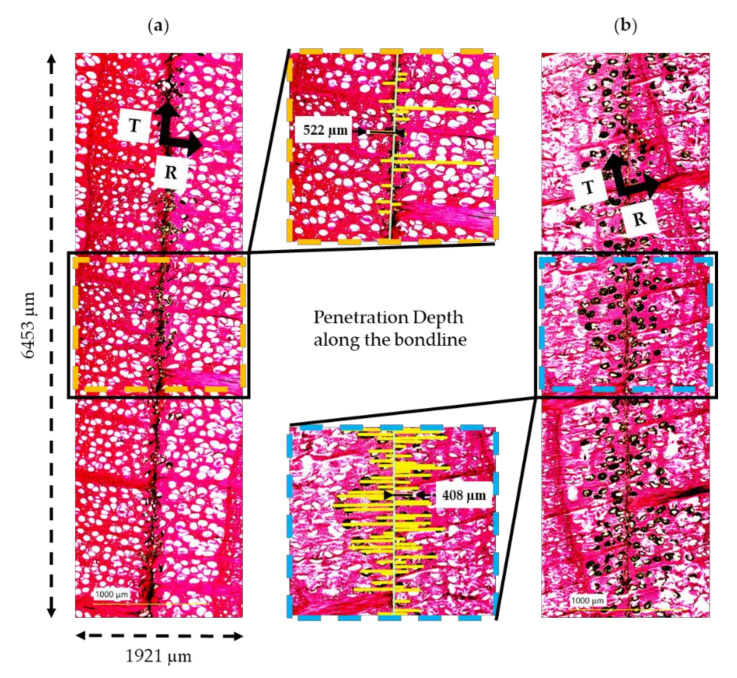
Images of adhesive penetration by brightfield microscopy on differently cut and anatomically almost identically aligned finger joints; the penetration depth of the adhesive in a predominantly radial direction is shown; a section (1921 × 6453 µm^2^) of the adhesive joints was examined via stitched images and the penetration was measured (yellow lines) perpendicular to the bondline; exemplary beech finger joints cut before bonding with (**a**) Cu1 (blunt) and F_R_1 (4 m min^−1^) and (**b**) Cu3 (sharp) and F_R_1 (4 m min^−1^) are shown.

For microscopic observation of the adhesive penetration into the wood surface, sample material was taken from the center of bonded finger joints that had previously been processed with the different cutting combinations. The bonding was carried out with a two-component MUF adhesive (mixing ratio 100:50; resin (MUF2): hardener) with an application quantity of 280 g m^−2^ and manual application, a pressing time of 5 s, and pressure of 12 N mm^−2^ on the finger-jointing machine according to the requirements of the adhesive manufacturer and EN 14080:2013 [33]. Several adhesive joints were localized on the cross-sectional surfaces of the sample material. For better visualization, the cross-sectional surfaces were first leveled with an automatic rotary microtome (Leica HistoCore AUTOCUT, Wetzlar, Germany) and stained with 0.5% safranin solution (Euromex, Arnhem, The Netherlands) for 15 min to increase contrast. In the following, the sample material was rinsed with demineralized water, and then a hot plate drying was carried out at 50 °C. To obtain a homogenous topography, the samples were cut again with the microtome, and thin sections (30 µm) were prepared. A fluorescence light microscope (microscope: BZ-X810, control unit: BZ-X800; Keyence Corporation, Osaka, Japan) was used to visualize the adhesive penetration in a predominantly radial direction (Figure 5). The adhesive used only fluoresced very weakly, so instead of fluorescence images, high-resolution brightfield images (1920 × 1440 px) were taken in colour mode. Initially, overview images of several adhesive joints were taken in the navigation mode, and a representative adhesive joint section was selected for each cutting combination. The images were taken at 10× magnification with defined focal planes (Z-stack, upper and lower limit, pitch 2.5 µm) and, depending on the penetration of the adhesive, individual images in 6 × 1 (1921 × 6451 µm^2^) and 6 × 2 (2513 × 4985 µm^2^) were automatically merged to form an overall image. The processing and analysis of the images were carried out with the BZ-X Analyzer (Keyence Corporation, Osaka, Japan), and the penetration was determined perpendicular to the adhesive joint with the measuring function XY measurement (perpendicular line). The bondlines were almost exclusively in the earlywood. All vessels were considered that were at least partially filled with adhesive and could be clearly identified in the interface zone between the adhesive and the wood joining parts (Figure 5). All other wood anatomical structures that might be involved in adhesive migration, like wood rays [30], were not considered in the measurements.

#### 2.3.4. Data Processing

To evaluate the effects and the interactions of the parameter settings

- wood, cutter sharpness, feed rate, and adhesive resp. early-/latewood on the resulting surface parameters
- contact angle (wettability), roughness, and penetration depth (penetrability), a statistic test design with three Full Factorial Designs was set up (Table 2).

This results in 24 test points for the response variables contact angle and roughness. Since the penetration depth measurements were carried out with only one type of adhesive, the design here consists of only 12 test points. It should be noted that in the case of the roughness measurements on birch, no distinction between early- and latewood was found. For this reason, the design contains a constant setting of the factor level early-/latewood (E/L). The number of measurement replicates per response variable is given in Table 2. The results are presented below using boxplots, 2-way interaction plots, and scatter plots with linear function (linear fit).

**Figure 6:**
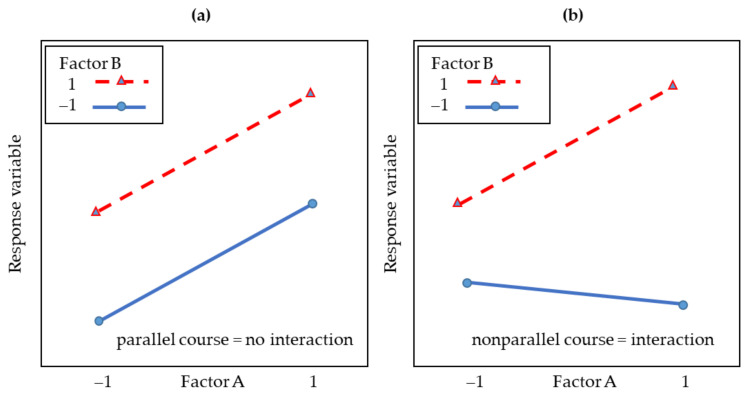
Own illustration of interactions according to [39,40]; mean values of the factors are shown; the degree of nonparallelism of the effect lines is an indicator of the intensity of the interaction. If the effect lines are parallel (**a**), there is no interaction. If they are nonparallel (**b**), there is an interaction.

The interaction plots represent the mean values of all settings of one factor as a function of the setting of another factor. Figure 6 shows how interactions can be detected. If the straight lines are approximately parallel, only a small interaction exists [39]. The scatter plots are intended to show how the individual response variables relate to each other. The significance of the main effects and interactions was tested for all three Full Factorial Designs using an ANOVA [40]. The significance level was set to the value of 0.05. Non-significant effects are highlighted in red and marked non-sig in the 2-way interaction plots.

## 3. Results and Discussion

**Figure 7:**
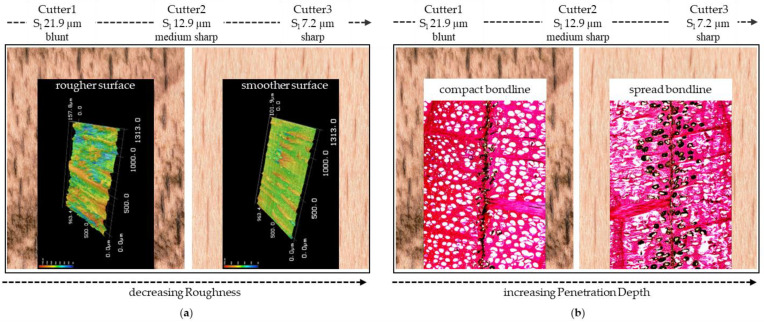
Resulting wood surfaces for beech after processing with low feed rate (F_R_1, 4 m min^−1^) and different sharp cutters (S_l_; microscopically measured cutting-edge radius); trend observed change in roughness, the blunt cutter (Cu1) resulted in a rougher surface and the sharp cutter (Cu3) resulted in a smoother surface (**a**), and the trend observed change in penetration depth, the blunt cutter (Cu1) resulted in a compact bondline and the sharp cutter (Cu3) resulted in a spread bondline (**b**); the surfaces machined with a low feed rate and a blunt cutter showed a charring of the wood surface.

In this study, wood cutting surfaces were prepared, which in some cases were recognizably different from each other to the naked eye or by manual palpation. The cutting combination of a blunt cutter 1 (Cu1) and slow feed rate 1 (F_R_1) led to a charring of the wood surface, which was particularly visible on the higher-density beech. Using contact angle measurements and microscopic methods, less obvious differences in the cutting surfaces were detected, and effects and interactions will be discussed in the following. In Figure 7, the trend of decreasing roughness and increasing adhesive penetration with increasing cutter sharpness observed in this study comparable for both wood species is shown exemplarily for the beech. The mean cutting-edge radius of the blunt cutter Cu1 was 21.9 µm, that of the medium-sharp cutter Cu2 was 12.9 µm, and that of the sharp cutter Cu3 was 7.2 µm.

### 3.1. Main Effects and 2-Way Interactions

**Figure 8:**
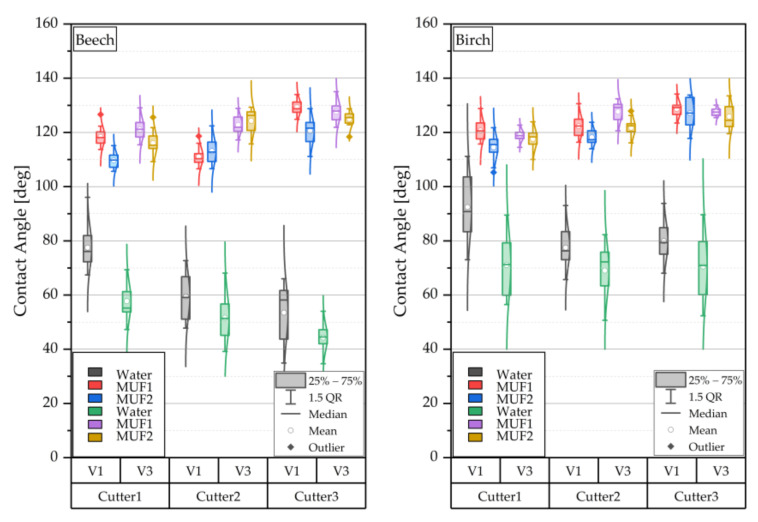
Effects on contact angle after water application and different viscous MUF-resins; Cu1 blunt, Cu2 medium sharp, and Cu3 sharp; F_R_1 4 m min^−1^ and F_R_2 25 m min^−1^.

Figure 8 shows the distributions of the measured values from the contact angle determination for the three cutter sharpness levels and the two feed rates examined—beech on the left and birch on the right. In principle, the measured values are approximately normally distributed around the mean value for all factor settings. For both wood species, a decreasing contact angle is observed with increasing cutter sharpness in the case of the test liquid water. For the two adhesives tested, MUF1 and MUF2, an increasing contact angle is observed with increasing cutter sharpness, and the change is much less pronounced than for the water. The measurements of the test liquid water generally show significantly greater scatter than those of the two adhesives. Water resulted in lower contact angles at all degrees of cutter sharpness for the setting of a higher feed rate compared to a lower feed rate setting. This trend does not exist when looking at the two adhesives. A higher feed rate led to higher contact angles. When comparing the two adhesive systems, the adhesive with the lower viscosity MUF2 (same density as MUF1) has a lower position of the measured distribution of the contact angle. It is not clear whether only the rate of the wetting process or the contact angle values themselves were influenced by the different viscosity [41]. The contact angles measured with water were overall higher for birch than for beech, which may be explained by the higher content of hydrophobic wood extractives in birch [42]. Lower contact angles of the applied liquid signify better wetting of the surface, and this is an important requirement for the adhesion of wood bonded joints and their performance [43,44]. The higher contact angles of the water with decreasing sharpness of the cutter and feed rate indicate that the wood surface became more hydrophobic. Chemical and physical effects caused by the cutting process (comparable effects that can occur during wood modification, activation, and functionalization of wood surfaces) can be the reason for a changed contact angle (wettability) [45]. The formation of polar or functional groups caused by the cutting process of the wood surface (altered oxygen-carbon ratios and polarity) could have led to an altered wetting process at the interface between wood and water/adhesive. Regarding this, altered molecular-physical interactions between the molecules of the liquids and the functional groups of the cut wood surfaces have to be investigated in more detail, which was attempted partially in this study. However, it was not possible to determine the surface free energy at the end-grain wood, as the non-polar test liquid (diiodomethane), usually used for this purpose in combination with water, penetrates too fast into the wood structure, even after reconditioning the wood samples. A determination of the surface free energy of the adhesive resins (dispersive and polar components) would be the next step to better explain the interactions. To detect functional groups on the cut wood surfaces, Fourier-transform infrared spectroscopy (FTIR) [46] and X-ray photoelectron spectroscopy (XPS) is commonly used.

**Figure 9:**
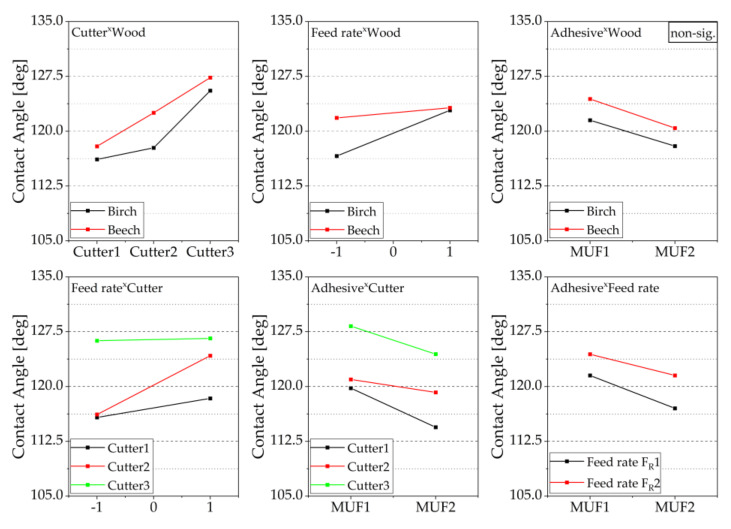
2-way interactions of the contact angle for different cutting combinations, mean values of the factors are shown, interactions are visualized based on the courses of the effect lines; please see explanatory Figure 6.

The interaction diagram (Figure 9) shows that the strongest effect on forming the contact angle between the wood surface and the adhesive is caused by the cutter sharpness for beech and birch. Increasing the feed rate has a stronger effect on birch than on beech. For low feed rates, there are large differences in the measured contact angles between the two wood species. In the case of the higher feed rate, similarly large values are found. In general, an increase in the feed rate leads to higher contact angles. Only a slight interaction between adhesive and wood species is observed, which is not significant. In general, it can be determined that the adhesive with the lower viscosity (MUF2) results in lower contact angles for both wood species. Regardless of the variation of the other parameters, it can be seen from the interaction diagram that the higher contact angles were measured for the beech. A strong interaction between feed rate and cutter can only be observed for Cu2. However, a higher feed rate causes higher contact angles for all three cutters. A stronger interaction between adhesive and cutter can also only be observed for Cu2. The influence of the feed rate is also very similar for both adhesives.

Different contact angles could be explained by the physical influence of roughness, which varied depending on the cutting combination (Figure 8). In this study, the contact angle measured with water increased with increasing roughness. In [47], where contact angle measurements were performed on wood surfaces after sanding with different grit sizes, a similar relation between water contact angle and roughness was found. The relation shown in the present study was opposite for the adhesive and confirmed the results obtained by [16], who found that the contact angle of the tested adhesives decreases with increasing roughness. It is unclear whether physical or chemical effects are dominant because of certain cutting combinations and possibly interact with each other.

**Figure 10:**
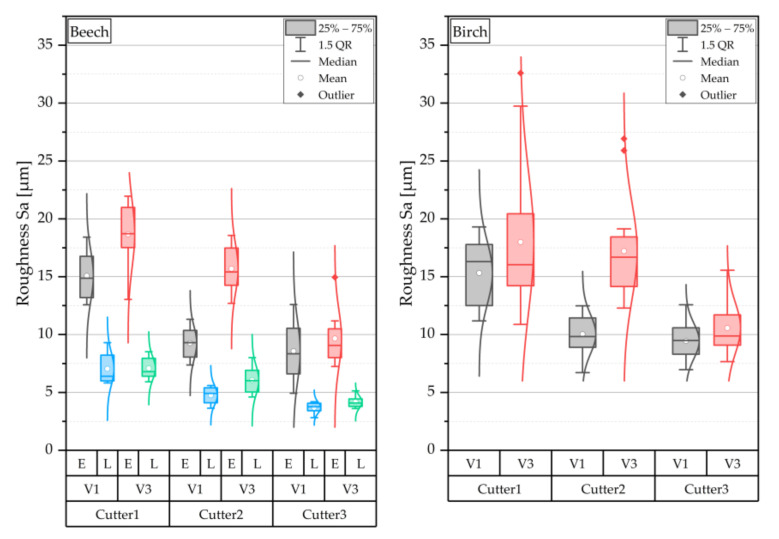
Effects on roughness and differentiation of the roughness of beech early (E)- and latewood (L); Cu1 blunt, Cu2 medium sharp, and Cu3 sharp; F_R_1 4 m min^−1^ and F_R_2 25 m min^−1^.

**Figure 11:**
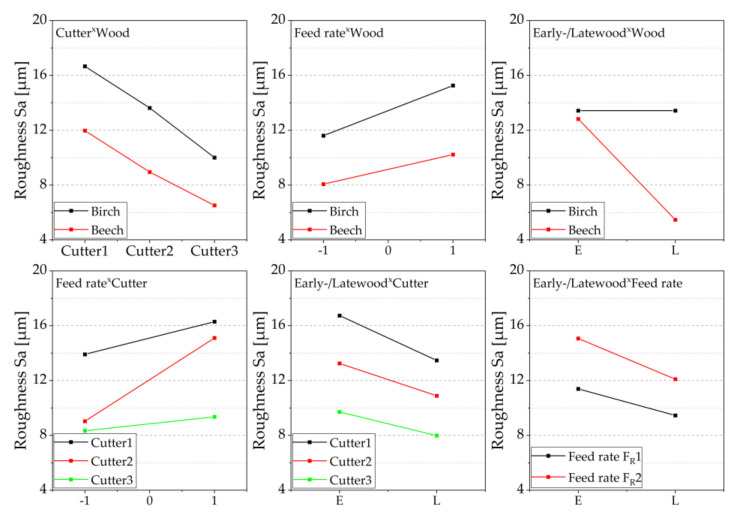
2-way interactions of the roughness for different cutting combinations, mean values of the factors are shown, and interactions are visualized based on the courses of the effect lines; please see explanatory Figure 6.

In analogy to the presented results of the contact angle measurements, Figure 10 shows the effects of the test settings on the target parameter roughness. For the birch, the diagram does not distinguish between early- and latewood. The measurements of both wood species showed that an increasing degree of sharpness of the cutter leads to lower mean roughness (Sa). This was also observed in [14], where the mechanical stress on the wood surface was higher with blunter tools and lower with sharper tools. Sharp planing knives produced clear and smooth cut edges with small and few cell fragments. In contrast, blunt planing knives produced fibrous and rougher cut edges with larger and multiple cell fragments. Likewise, it can be seen for both wood species that a higher feed rate leads to an increase in the evaluated surface roughness value (Sa). As a tendency, a sharper cutter causes a lower scatter of the surface roughness. This also tends to be observed for the lower feed rate. The average Sa roughness of birch was higher than that of beech. The reasons for that might be associated with differences in the anatomical structure (e.g., size and number of vessels) and the lower density of birch [22]. The effect of early- and latewood on roughness was very pronounced for beech, with earlywood showing significantly higher roughness (Figure 11). This has been described previously for other wood species [48,49]. For further characterization of the cutting surface, in addition to the Sa roughness parameter measured in this study, hybrid parameters such as the Sdr (developed interfacial area ratio) can be used. This is closely related to the bonding quality, and it defines the topographic magnification of the real surface related to the size of the two-dimensional projected measuring area [37]. Among others, [50] found that the bond strength decreases with increasing roughness of the wood surface. Possible reasons should be discussed, as on the one hand rougher surface can be expected to provide better mechanical anchorage of the adhesive. On the other hand, a smoother surface may allow a closer interaction of wood and adhesive molecules and thus greater adhesion forces.

Figure 11 shows the interaction diagram for the investigated target parameter roughness. The roughness decreases with increasing cutter sharpness, and the course of the effect lines is almost parallel. There is, therefore, no interaction between wood species and cutter sharpness. The adjustment of the cutter sharpness had a similar effect on both wood species. For birch, which did not allow a distinction between early- and latewood, higher roughness was found, regardless of the other factors. The feed rate also interacts only very slightly with the wood species. Higher feed rates lead to higher roughness for both species. Since there was no differentiation between early- and latewood in the case of birch, this effect can only be shown for beech. The diagram shows, however, that this effect is very pronounced and that there is a large difference between the average roughness in the early- and latewood. The average roughness of the latewood is significantly lower, as already shown in the boxplot (Figure 10). The interaction between feed rate and cutter shows, as with the interaction of the contact angle, that Cu2 interacts more strongly. Higher feed rates lead to increasing roughness—this was particularly the case with Cu2. Almost independently of the cutter sharpness and the feed rate, lower average roughness is found for latewood, whereby only the wood species beech was included here.

**Figure 12:**
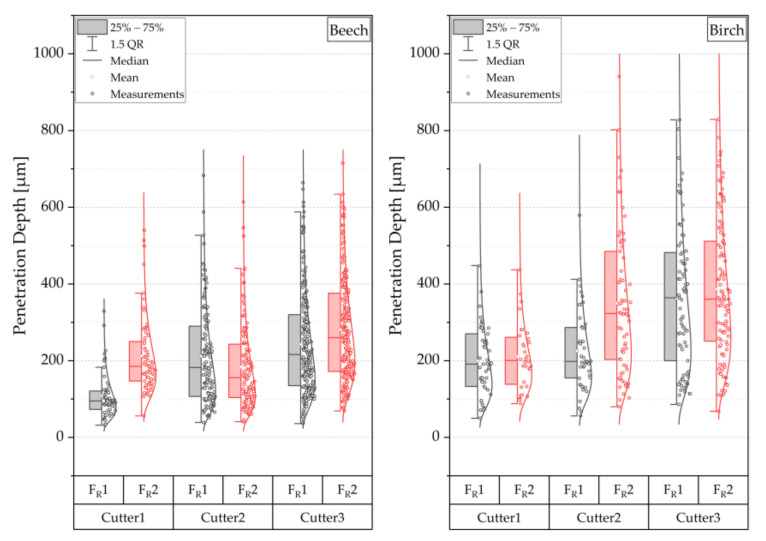
Effects on penetration depth and visualization of the distribution of vessels filled with adhesive; Cu1 blunt, Cu2 medium sharp, and Cu3 sharp; F_R_1 4 m min^−1^ and F_R_2 25 m min^−1^.

**Figure 13:**
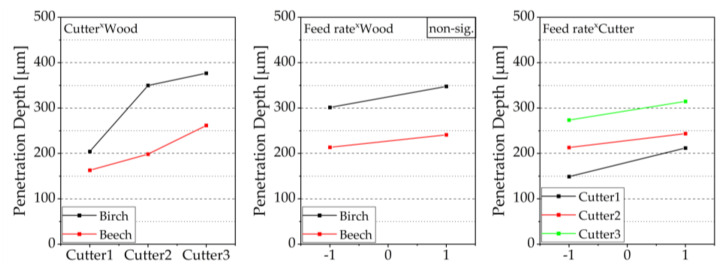
2-way interactions of the penetration depth for different cutting combinations, mean values of the factors are shown, interactions are visualized based on the courses of the effect lines; please see explanatory Figure 6.

Figure 12 shows the distributions of the measured penetration depths with the varying factor-value combinations. In addition, the distributions of the measured values are shown using points and a fit to a log-normal distribution, which is a good approximation of the distributions. In general, the beech showed lower penetration depths. As the cutter sharpness increases, greater adhesive penetration depths are measured. The feed rate shows only a weak interaction with the wood species (Figure 13). Generally, a higher feed rate causes greater adhesive penetration for all three cutters and both wood species. The beech had considerably more smaller vessels filled with adhesive, and the scattering of the penetration depth was less than the birch.

Contrary to the results of [51], where a smaller contact angle, resulting from lower viscosity and rougher surface, produced deeper penetration, while a larger contact angle, resulting from higher viscosity and smoother surface, produced shallower penetration, the adhesive penetration in this study increased with increasing contact angle and decreased with increasing roughness. A reason for this could be that at higher contact angles, more adhesive remains on the wood surface before pressing the glued wood joining parts than at lower contact angles and better wood surface wetting. Consequently, at higher contact angles, there is more adhesive on the wood surface that can be pressed into deeper lying wood structures. If a bigger amount of adhesive has already penetrated itself at lower contact angles before applying pressure, there is less adhesive on the wood surface that can be pressed into the wood structure. This may need to be investigated further. It can be assumed that the sharp cutter Cu3 cut the wood surface smoother (less roughness, Figure 10 and Figure 11) and thus enabled better penetration of the adhesive into the intact wood structures [15]. It is suspected that the blunter cutter resulted in greater damage to the wood surface due to the mechanical stress and consequent compaction of the penetration paths. This effect was possibly intensified by the low feed rate combined with the blunt Cu1 (charring of the wood surface, Figure 7). Usually, the mechanical stress on the wood surface is higher at higher feed rates [52]. To further check the effects, scanning electron microscopy (SEM) images of the first cell layers of the wood surfaces as made in [14] are recommended.

The brightfield microscopy method used here is a simplified way of visualizing adhesive penetration in a 2D plane of observation. In various studies (e.g., [30,32]), adhesive penetration was tested using 3D methods (e.g., micro-computed tomography), which depicts the complexity of adhesive penetration into the wood structure more reliably than 2D methods, in particular, when hardwoods with their complex anatomical structures are considered. In this study, various layers of the bonded wood samples were considered in preliminary tests, and representative bondline sections were selected for each cutting combination using the navigation mode of the fluorescence light microscope. This seems to be sufficiently accurate for a first comparison of the cutting combinations. If 3-D microscopy methods are available, they should be used in preference, whereby a considerably more time-intensive scanning and analysis must be considered. However, researchers should be aware that micro-computed tomography often requires tagging the adhesive with an X-ray dense mark to make them visible in 3D tomography images [53,54,55].

### 3.2. Interactions of the Surface Parameters and Results of ANOVA

**Figure 14:**
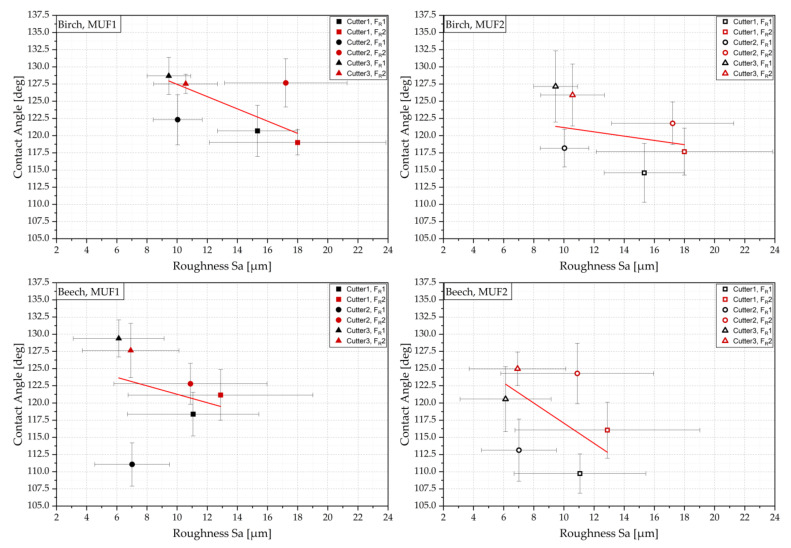
Interactions between contact angle and roughness for different cutting combinations; red line shows trendline and error bars show standard deviation in x (roughness) and y (contact angle) direction.

Figure 14 shows the mean values of the cutting combinations with their respective standard deviation over the roughness and the contact angle and visualizes the trend by a linear fit. In general, it can be observed that the contact angles determined decrease with increasing roughness. In the case of Cu3, which gave low roughness for both wood species and both adhesives, high contact angles are shown. In the case of Cu1, the roughnesses are clearly above those found on Cu3. Here, the contact angle measurement resulted in the lowest contact angles. The mean contact angle of Cu2 is in between but partly deviated stronger from the trend. However, a correlation between feed rate, roughness, and contact angle cannot be clearly identified.

**Figure 15:**
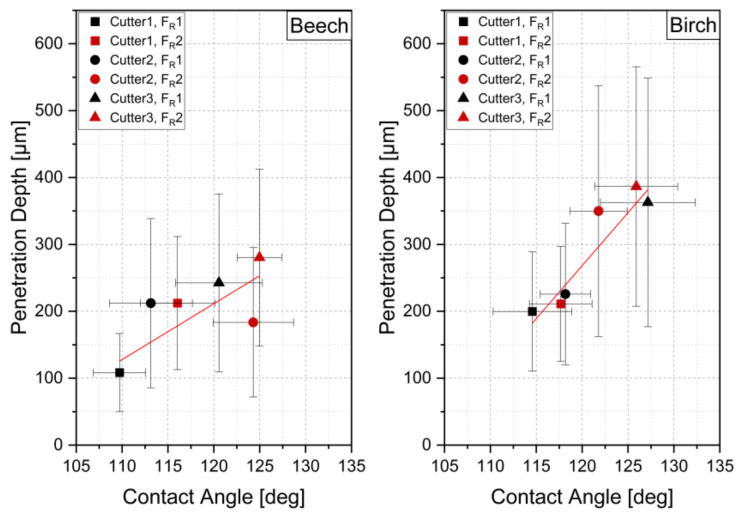
Interactions between penetration depth and contact angle for different cutting combinations; the red line shows trendline, and error bars show standard deviation in x (contact angle) and y (penetration depth) direction.

The interaction between contact angle and penetration depth is shown in Figure 15. It shows that the penetration depth increases with increasing contact angle for both wood species. In particular, the mean values for the cutters Cu1 and Cu3 clearly show this trend for birch, while Cu2 simultaneously shows a clear effect of the cutting feed rate.

**Figure 16:**
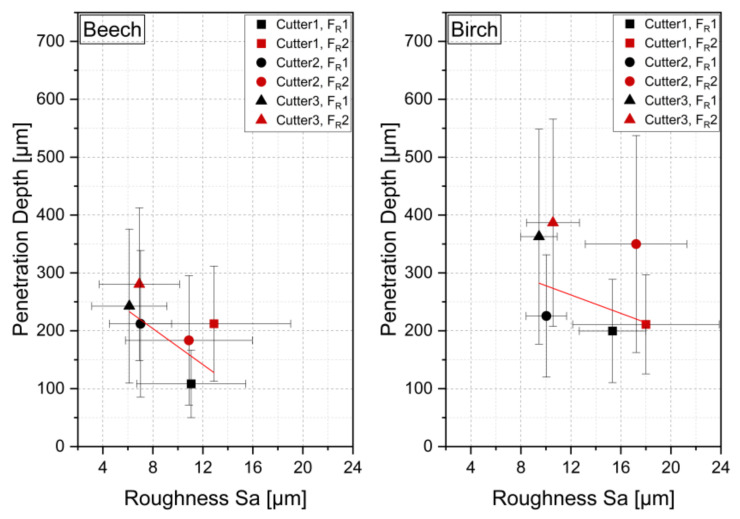
Interactions between penetration depth and roughness for different cutting combinations; the red line shows trendline, and error bars show standard deviation in x (roughness) and y (penetration depth) direction.

The interaction between roughness and penetration depth for the different settings is shown in Figure 16. As the mean roughness increases, a lower penetration depth is obtained. The sharpest cutter, Cu3, showed the lowest roughness values and enabled the deepest adhesive penetration. As the cutter sharpness decreases, the roughness increases and the penetration depth decreases. This trend can be observed for both wood species. For birch, where higher roughness was measured, a greater penetration depth was found on average.

The significance of the main effects and interactions tested for all three Full Factorial Designs using an ANOVA is shown in Table 3. The significance level was set to the value of 0.05. Non-significant effects are highlighted in red and marked non-sig within the interaction plot concerned. A non-significant result was obtained from the analysis of variance for the interaction between wood and adhesive on the contact angle and for the interaction between wood and feed rate on the adhesive penetration. All other interactions, as well as the main effects, are significant at the predefined level of 0.05.

## 4. Conclusions

The changes in cutting hardwood finger joints caused by cutter sharpness level and feed rate have significant consequences for the wood surface parameters wettability, roughness, and penetrability. The present study, whose analysis is based on non-destructive methods and three Full Factorial Designs, leads to the following conclusions:

Main results:

- Effects were caused by the cutter sharpness, and the two investigated feed rates rather caused an enhancing effect on the surface parameters.
- The wood species (beech and birch) behaved quite similarly overall in terms of the observed effects, which was expected as they have a similar wood density and anatomy. Considering anatomical differences in the study is essential to show and compare effects and interactions.
- The cutting wood surfaces had partly opposite effects on different wetting liquids, shown with water and adhesive resin. A new finding is that higher contact angles of adhesives occur with sharper cutters.

Perspectives:

- Further chemical investigations of the wood surface are necessary, such as FTIR and XPS. Microscopy methods with stitching function used in this study were well suited to view large observation areas in high resolution. Future studies could consider 3D microscopy providing additional information on the complex adhesive penetration into hardwoods.
- The present study provided the first examinations of the surface design of finger-jointed hardwoods and shows options for evaluating the wood surface quality after machining. The question of what a machined wood surface must look like (surface design) to enable efficient bonding needs further research attention. Further tests on bonding strength and resistance can follow up to evaluate the effect of the cutting surface on the bonding performance.

## Figures and Tables

**Table 1:** Basic information on the contact angle measurement for the evaluation of wettability.

Test Liquid	Density [g cm^−3^] ^1^ Viscosity [mPa s]	Device	Method
Water	1.0 0.9 (at 23 °C)	Mobile Surface Analyzer One-Click SFE	Automatic dosing with 1 µL, Fitmethod Ellipse (Tang.-1)
Melamine-Urea-Formaldehyde resin 1 (MUF1)	1.27 10,000–25,000 (at 25 °C)	Drop Shape Analyzer 100	Manual syringe dosing, Fitmethod Ellipse (Tang.-1)
Melamine-Urea-Formaldehyde resin 2 (MUF2)	1.28 2000–3500 (at 20 °C)	Drop Shape Analyzer 100	Manual syringe dosing, Fitmethod Ellipse (Tang.-1)

^1^ Information on water from Krüss GmbH and on adhesive resins from the adhesive manufacturer’s datasheets.

**Table 2:** Parameters, settings, and Full Factorial Design.

(a) contact angle with 24 parameter settings					
**Wood (W)**	**Cutter (Cu)**	**Feed rate (F_R_)**	**Adhesive (A)**		
Beech	Cutter1	F_R_1 (1)	MUF1		
Birch	Cutter2	F_R_2 (−1)	MUF2		
	Cutter3				
**No.**	**N**	**W**	**Cu**	**F_R_**	**A**
1	16	Beech	Cu1	F_R_1 (−1)	MUF1
2	16	Beech	Cu2	F_R_1 (−1)	MUF1
3	13	Beech	Cu3	F_R_1 (−1)	MUF1
4	14	Beech	Cu1	F_R_2 (1)	MUF1
5	16	Beech	Cu2	F_R_2 (1)	MUF1
6	16	Beech	Cu3	F_R_2 (1)	MUF1
7	16	Beech	Cu1	F_R_1 (−1)	MUF2
8	16	Beech	Cu2	F_R_1 (−1)	MUF2
9	15	Beech	Cu3	F_R_1 (−1)	MUF2
10	16	Beech	Cu1	F_R_2 (1)	MUF2
11	15	Beech	Cu2	F_R_2 (1)	MUF2
12	15	Beech	Cu3	F_R_2 (1)	MUF2
13	15	Birch	Cu1	F_R_1 (−1)	MUF1
14	16	Birch	Cu2	F_R_1 (−1)	MUF1
15	16	Birch	Cu3	F_R_1 (−1)	MUF1
16	15	Birch	Cu1	F_R_2 (1)	MUF1
17	16	Birch	Cu2	F_R_2 (1)	MUF1
18	16	Birch	Cu3	F_R_2 (1)	MUF1
19	16	Birch	Cu1	F_R_1 (−1)	MUF2
20	16	Birch	Cu2	F_R_1 (−1)	MUF2
21	16	Birch	Cu3	F_R_1 (−1)	MUF2
22	16	Birch	Cu1	F_R_2 (1)	MUF2
23	14	Birch	Cu2	F_R_2 (1)	MUF2
24	11	Birch	Cu3	F_R_2 (1)	MUF2
(b) roughness with 24 parameter settings					
**Wood (W)**	**Cutter (Cu)**	**Feed rate (F_R_)**	**E-/L-wood (E/L)**		
Beech	Cutter1	F_R_1 (−1)	E (E/L)		
Birch	Cutter2	F_R_2 (1)	L (E/L)		
	Cutter3				
**No.**	**N**	**W**	**Cu**	**F_R_**	**E/L**
1	8	Beech	Cu1	F_R_1 (−1)	E
2	8	Beech	Cu2	F_R_1 (−1)	E
3	8	Beech	Cu3	F_R_1 (−1)	E
4	8	Beech	Cu1	F_R_2 (1)	E
5	8	Beech	Cu2	F_R_2 (1)	E
6	8	Beech	Cu3	F_R_2 (1)	E
7	8	Beech	Cu1	F_R_1 (−1)	L
8	8	Beech	Cu2	F_R_1 (−1)	L
9	8	Beech	Cu3	F_R_1 (−1)	L
10	8	Beech	Cu1	F_R_2 (1)	L
11	8	Beech	Cu2	F_R_2 (1)	L
12	8	Beech	Cu3	F_R_2 (1)	L
13	8	Birch	Cu1	F_R_1 (−1)	E/L
14	8	Birch	Cu2	F_R_1 (−1)	E/L
15	8	Birch	Cu3	F_R_1 (−1)	E/L
16	8	Birch	Cu1	F_R_2 (1)	E/L
17	8	Birch	Cu2	F_R_2 (1)	E/L
18	8	Birch	Cu3	F_R_2 (1)	E/L
19	8	Birch	Cu1	F_R_1 (−1)	E/L
20	8	Birch	Cu2	F_R_1 (−1)	E/L
21	8	Birch	Cu3	F_R_1 (−1)	E/L
22	8	Birch	Cu1	F_R_2 (1)	E/L
23	8	Birch	Cu2	F_R_2 (1)	E/L
24	8	Birch	Cu3	F_R_2 (1)	E/L
(c) penetration depth with 12 parameter settings					
**Wood (W)**	**Cutter (Cu)**	**Feed rate (F_R_)**	**Adhesive (A)**		
Beech	Cutter1	F_R_1 (1)	MUF2		
Birch	Cutter2	F_R_2 (−1)			
	Cutter3				
**No.**	**N**	**W**	**Cu**	**F_R_**	**A**
1	59	Beech	Cu1	F_R_1 (−1)	MUF2
2	133	Beech	Cu2	F_R_1 (−1)	MUF2
3	221	Beech	Cu3	F_R_1 (−1)	MUF2
4	65	Beech	Cu1	F_R_2 (1)	MUF2
5	122	Beech	Cu2	F_R_2 (1)	MUF2
6	227	Beech	Cu3	F_R_2 (1)	MUF2
7	47	Birch	Cu1	F_R_1 (−1)	MUF2
8	44	Birch	Cu2	F_R_1 (−1)	MUF2
9	77	Birch	Cu3	F_R_1 (−1)	MUF2
10	32	Birch	Cu1	F_R_2 (1)	MUF2
11	69	Birch	Cu2	F_R_2 (1)	MUF2
12	108	Birch	Cu3	F_R_2 (1)	MUF2

**Table 3:** ANOVA results table (sig. level 0.05) based on the Full Factorial Designs from Table 2.

	Contact Angle ANOVA—[a]		Roughness ANOVA—[b]		Penetration Depth ANOVA—[c]	
Main Effect/ 2W-Interaction	F-Value	Prob > F	F-Value	Prob > F	F-Value	Prob > F
Wood	52.011	3.589 × 10^−12^	128.642	1.491 × 10^−24^	135.650	1.031 × 10^−29^
Cutter	204.809	3.764 × 10^−59^	102.813	9.558 × 10^−34^	70.457	1.369 × 10^−29^
Feed rate	96.586	3.106 × 10^−20^	78.592	1.164 × 10^−16^	14.017	1.901 × 10^−4^
Adhesive	82.063	1.043 × 10^−17^	47.373	4.261 × 10^−11^	-	-
Wood × Cutter	7.034	0.001	1.118	0.328	6.44502	0.00165
Wood × Feed rate	33.083	1.963 × 10^−8^	3.909	0.049	0.14685 *	0.70163 *
Wood × Adhesive	0.001 *	0.971 *	94.746	2.484 × 10^−19^	-	-
Cutter × Feed rate	36.010	6.477 × 10^−15^	18.029	4.575 × 10^−8^	9.102	1.195 × 10^−4^
Cutter × Adhesive	8.533	2.419 × 10^−4^	1.583	0.207	-	-
Feed rate × Adhesive	6.552	0.010	2.103	0.148	-	-

* non-sig.

## Data Availability

Not applicable.

## References

[1] Polley H. (2018). Der Wald in Deutschland: Ausgewählte Ergebnisse Der Dritten Bundeswaldinventur.

[2] Weimar H., Jochem D. (2013). Holzverwendung im Bauwesen—Eine Marktstudie im Rahmen der “Charta für Holz”.

[3] Knauf M., Frühwald A. (2020). Laubholz-Produktmärkte Aus Technisch-Wirtschaftlicher Und Marktstruktureller Sicht.

[4] Obernostererer D., Jeitler G., Schickhofer G. (2022). Birke: Holzart der Zukunft im Modernen Holzbau.

[5] Informationsverein Holz e.V. (2017). Konstruktive Bauprodukte Aus Europäischen Laubhölzern.

[6] Linsenmann P. (2016). European Hardwoods for the Building Sector (EU Hardwoods).

[7] Wehrmann W., Torno S. (2015). Laubholz für tragende Konstruktionen—Zusammenstellung zum Stand von Forschung und Entwicklung.

[8] Ehrhart T. (2019). European Beech—Glued Laminated Timber.

[9] Aicher S. (2014). Geklebte Verbindungen in Holzbauprodukten Und—Tragwerken.

[10] Timbolmas C., Rescalvo F.J., Portela M., Bravo R. (2022). Analysis of Poplar Timber Finger Joints by Means of Digital Image Correlation and Finite Element Simulation Subjected to Tension Loading. Eur. J. Wood Prod..

[11] Krackler V., Keunecke D., Niemz P. (2010). Verarbeitung und Verwendungsmöglichkeiten von Laubholz: Entscheidungsgrundlagen Zur Förderung Von Laubholzverarbeitung Und—Absatz.

[12] Volkmer T., Lehmann M., Clerc G. (2017). Brettschichtholz Aus Buche: Keilzinkenverbindung Und Flächenverklebung.

[13] Fortuna B., Azinović B., Plos M., Šuligoj T., Turk G. (2020). Tension Strength Capacity of Finger Joined Beech Lamellas. Eur. J. Wood Prod..

[14] Lütkemeier B. (2018). Kleben Von modifiziertem Vollholz—Gestaltung Des Grenzbereichs Zur Steuerung Der Verklebungsmechanismen.

[15] Röver D. (2020). Entwicklung Neuartiger Knotenverstärkungen Von Holztragwerken Mit Kunstharzpressholz (KP).

[16] Yorur H., Erer A.M., Oğuz S. Effect of Surface Roughness on Wettability of Adhesive on Wood Substrates. Proceedings of the 3rd International Conference on Science, Ecology and Technology.

[17] Petrič M., Oven P. (2015). Determination of Wettability of Wood and its Significance in Wood Science and Technology: A Critical Review. Rev. Adhes. Adhes..

[18] Qin Z., Gao Q., Zhang S., Li J. (2014). Surface Free Energy and Dynamic Wettability of Differently Machined Poplar Woods. BioResources.

[19] Gindl M., Reiterer A., Sinn G., Stanzl-Tschegg S.E. (2004). Effects of Surface Ageing on Wettability, Surface Chemistry, and Adhesion of Wood. Holz. Roh. Werkst..

[20] Shi S.Q., Gardner D.J. (2001). Dynamik Adhesive Wettability of Wood. Wood Fiber Sci..

[21] Gurau L. (2022). Testing the Processing-Induced Roughness of Sanded Wood Surfaces Separated from Wood Anatomical Structure. Forests.

[22] Jankowska A. (2020). Understanding of Surface Roughness of Wood Based on Analysis its Structure and Density. Ann. WULS For. Wood Technol..

[23] Sandak J., Orlowski K.A., Sandak A., Chuchala D., Taube P. (2020). On-Line Measurement of Wood Surface Smoothness. Drv. Ind..

[24] Qing L., Li Z.F., Xing D. (2018). Study on Evaluation Method of Surface Roughness of Wood Processing. Proceedings of the International Workshop on Materials, Chemistry and Engineering.

[25] De Conti A.C., de Conti C. (2015). Effect of Surface Roughness on the Shear and Tensile Strength of Hardwood Adhesive Joints: A Linear Elastic Model. Adv. Mater. Res..

[26] Sinn G., Sandak J., Ramananantoandro T. (2009). Properties of Wood Surfaces—Characterisation and Measurement. A Review COST Action E35 2004–2008: Wood Machining—Micromechanics and Fracture. Holzforschung.

[27] Li W., Zhang Z., Mei C., Kibleur P., Van Acker J., Van Den Bulcke J. (2022). Understanding the Mechanical Strength and Dynamic Structural Changes of Wood-Based Products Using X-ray Computed Tomography. Wood Mater. Sci. Eng..

[28] Shirmohammadi M., Leggate W. (2021). Review of Existing Methods for Evaluating Adhesive Bonds in Timber Products. Engineered Wood Products for Construction.

[29] Park S., Jeong B., Park B.-D. (2021). A Comparison of Adhesion Behavior of Urea-Formaldehyde Resins with Melamine-Urea-Formaldehyde Resins in Bonding Wood. Forests.

[30] Bastani A., Adamopoulos S., Koddenberg T., Militz H. (2016). Study of Adhesive Bondlines in Modified Wood with Fluorescence Microscopy and X-Ray Micro-Computed Tomography. Int. J. Adhes. Adhes..

[31] Volkmer T., Franke B., Schusser A. Analysis of the Penetration of Adhesives at Finger-Joints in Beech Wood. Proceedings of the World Conference on Timber Engineering 2014.

[32] Hass P., Wittel F.K., Mendoza M., Herrmann H.J., Niemz P. (2012). Adhesive Penetration in Beech Wood: Experiments. Wood Sci. Technol..

[33] (2013). Timber Structures—Glued Laminated Timber and Glued Solid Timber—Requirements.

[34] Oberhofnerová E., Pánek M. (2016). Surface Wetting of Selected Wood Species by Water during Initial Stages of Weathering. Wood Res..

[35] Wålinder M.E.P., Johansson I. (2001). Measurement of Wood Wettability by the Wilhelmy Method. Part 1. Contamination of Probe Liquids by Extractives. Holzforschung.

[36] Bastani A., Adamopoulos S., Militz H. (2015). Water Uptake and Wetting Behaviour of Furfurylated, N-Methylol Melamine Modified and Heat-Treated Wood. Eur. J. Wood Wood Prod..

[37] (2016). Geometrical Product Specifications (GPS)—Surface Texture: Areal—Part 1: Indication of Surface Texture.

[38] (2015). Geometrical Product Specifications (GPS)—Filtration—Part 1: Overview and Basic Concepts.

[39] Technical Statistics—Experimental Methodology (DoE) (2020). Quality Management in the Bosch-Group.

[40] Montgomery D.C. (2020). Design and Analysis of Experiments.

[41] Kúdela J. (2014). Wetting of Wood Surfaace by a Liquids of a Different Polarity. Wood Res..

[42] Papp E.A., Csiha C., Makk A.N., Hofmann T., Csoka L. (2020). Wettability of Wood Surface Layer Examined From Chemical Change Perspective. Coatings.

[43] Adamson A.W., Gast A.P. (1997). Physical Chemistry of Surfaces.

[44] Iždinský J., Reinprecht L., Sedliačik J., Kúdela J., Kučerová V. (2020). Bonding of Selected Hardwoods with PVAc Adhesive. Appl. Sci..

[45] Žigon J., Kovač J., Petrič M. (2022). The Influence of Mechanical, Physical and Chemical Pre-Treatment Processes of Wood Surface on the Relationships of Wood with a Waterborne Opaque Coating. Prog. Org. Coat..

[46] Zhou C., Jiang W., Cheng Q., Via B.K. (2015). Multivariate Calibration and Model Integrity for Wood Chemistry Using Fourier Transform Infrared Spectroscopy. J. Anal. Methods Chem..

[47] Papp E.A., Csiha C. (2017). Contact Angle as Function of Surface Roughness of Different Wood Species. Surf. Interfaces.

[48] Malkoçoğlu A. (2007). Machining Properties and Surface Roughness of Various Wood Species Planed in Different Conditions. Build. Environ..

[49] Csanády E., Magoss E. (2013). Mechanics of Wood Machining.

[50] Sogutlu C. (2017). Determination of the Effect of Surface Roughness on the Bonding Strength of Wooden Materials. BioResources.

[51] Cheng E., Sun X. (2006). Effects of Wood-Surface Roughness, Adhesive Viscosity and Processing Pressure on Adhesion Strength of Protein Adhesive. J. Adhes. Sci. Technol..

[52] Bustos C., Hernández R., Beauregard R., Mohammad M. (2004). Influence of Machining Parameters on the Structural Performance of Finger-Joined Black Spruce. Wood Fiber Sci..

[53] Modzel G., Kamke F.A., De Carlo F. (2011). Comparative Analysis of a Wood: Adhesive Bondline. Wood Sci. Technol..

[54] Paris J.L., Kamke F.A. (2015). Quantitative Wood–Adhesive Penetration with X-Ray Computed Tomography. Int. J. Adhes. Adhes..

[55] Paris J.L., Kamke F.A., Xiao X. (2015). X-Ray Computed Tomography of Wood-Adhesive Bondlines: Attenuation and Phase-Contrast Effects. Wood Sci. Technol..

